# Effective Treatment of Knee Osteoarthritis Using a Nano‐Enabled Drug Acupuncture Technology in Mice

**DOI:** 10.1002/advs.202302586

**Published:** 2023-08-09

**Authors:** Wenjie Xu, Yu Xiao, Minzhi Zhao, Jiahui Zhu, Yu Wang, Wenbin Wang, Peng Wang, Huan Meng

**Affiliations:** ^1^ CAS Key Laboratory for Biomedical Effects of Nanomaterials and Nanosafety and CAS Center for Excellence in Nanoscience National Center for Nanoscience and Technology Beijing 100190 China; ^2^ Beijing Hospital of Traditional Chinese Medicine Capital Medical University Beijing 100010 China; ^3^ University of Chinese Academy of Sciences Beijing 100049 China; ^4^ Chongqing University of Technology Chongqing 400054 China; ^5^ The First Affiliated Hospital of Zhengzhou University Zhengzhou Henan 450052 China

**Keywords:** acupuncture, knee osteoarthritis, lidocaine, nano drug delivery, nd‐Acu

## Abstract

A nano‐enabled drug delivery acupuncture technology (*nd*‐Acu) is developed that is based on traditional acupuncture needles where the stainless‐steel surface is designed to deliver various payload molecules. To create the *nd*‐Acu platform, an electrochemistry procedure is used to attach methyl salicylate‐modified cyclodextrin in which the sugar rings allow the encapsulation of structurally defined single or multiple payload molecules via an inclusion complexation process. Drug loading and release profile are first studied using fluorescent dyes abiotically and at intact animal level. *nd*‐Acu allows more efficient dye loading and time‐dependent release compared to pristine needles without cyclodextrin modification. Subsequently, a proof‐of‐principle efficacy study is conducted using the platform to load a local anesthetic, lidocaine, for the treatment of knee osteoarthritis (KOA) in mice. It is demonstrated that lidocaine‐laden *nd*‐Acu can effectively alleviate pain, reduce inflammation, and slow down KOA development biochemically and histologically. Hypothesis‐driven and proteomic approaches are utilized to investigate the working mechanisms of lidocaine *nd*‐Acu, indicating that the therapeutic outcome is attributed to the in vivo modulation of the HMGB1/TLR4 signaling pathway. The study also obtained preliminary evidence suggesting the involvement of mitochondria as well as small GTPase such as cdc42 during the treatment by lidocaine *nd*‐Acu.

## Graphical abstract

In this study, the authors successfully innovate a nano‐enabled drug delivery acupuncture technology (*nd*‐Acu) to enhance the therapeutic effect in a knee osteoarthritis (KOA) murine model. The comprehensive nano‐manufacture and biological data, combined with mechanistic exploration, experimentally demonstrate that *nd*‐Acu is a versatile nano‐enabled platform that can be used for implementing acupuncture and drug delivery concurrently to treat various diseases.

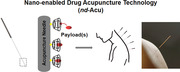

## 1 Introduction

Acupuncture is generally believed as “alternative medicine” in which fine needles are inserted into a particular area of the body. While the acupuncture theory and working mechanisms still require intensive investigation, certain applications (such as pain relief), although originated in China, are now practiced in the world such as the United States (US), European Union (EU), Australia, Japan, and Korea among other regions.^[^ ^1^ ^]^ Moreover, these acupuncture practices become popular research direction^[^ ^2^ ^]^ and are covered by major insurance plans.^[^ ^3^ ^]^

For pain relief or analgesic effects, the use of acupuncture or electro‐acupuncture is frequently associated with beneficial outcomes in various disease indications in both animals and humans. Take knee osteoarthritis (KOA) for example, this includes the demonstration of a retention rate of 77.8% in a small‐size, randomized pilot trial involving 36 participants.^[^ ^4^ ^]^ The long‐term effect of acupuncture for KOA patients is being investigated according to a published large clinical protocol that will determine the efficacy using the Western Ontario and McMaster Universities Osteoarthritis Index (WOMAC score).^[^ ^5^ ^]^ In 2022, an ongoing effort was to use meta‐analysis to summarize the analgesic effect based on the historical literature.^[^ ^6^ ^]^ For example, the benefit of acupuncture was linked to macrophage polarization in the inflamed KOA microenvironment, evidenced by reduced numbers of F4/80^+^CD86^+^ M1 macrophages and increased numbers of CD206^+^ M2 macrophages.^[^ ^7^ ^]^ Moreover, scientists revealed that electro‐acupuncture was capable of regulating endogenous opioid peptides to achieve analgesia outcomes.^[^ ^8^ ^]^ Noteworthy, acupuncture is commonly used in combination with Western medicine treatments such as medications and surgery to manage symptoms and side effects, and to enhance the effectiveness of Western medical treatments. This seems to be true in treating KOA because acupuncture can be used in conjunction with nonsteroidal anti‐inflammatory drugs (NSAIDs), corticosteroid injections, and hyaluronic acid injections, with proven evidence for improving joint function and slowing the disease progression.^[^ ^9^ ^]^ Interestingly, acupuncture point injection, which is a modified acupuncture technique in which liquid‐based API is injected into acupuncture point(s), has been applied to a wide range of diseases, particularly for pain control; this led to more than 60 clinical research in the last decade.^[^ ^10^ ^]^

Against this background, we hypothesize that using a nano‐enabled drug delivery acupuncture technology (*nd*‐Acu) will allow more effective treatment through complementary and combined acupuncture and drug delivery effects in a KOA murine model. Specifically, we developed an *nd*‐Acu platform through the use of an electrochemistry procedure to attach methyl salicylate‐modified cyclodextrin, allowing the encapsulation of single or multiple payload molecules as an inclusion complex.^[^ ^11^ ^]^ The first consideration for using *nd*‐Acu is the ability to combine treatment modalities, as shown by the efficient loading and release of fluorophores and APIs (e.g., lidocaine and non‐steroidal anti‐inflammatory drugs), together with the abovementioned acupuncture effect. The second consideration is that encapsulated nano delivery can improve PK and generate a local drug concentration at the acupuncture point. The third consideration is the ability to reduce toxicity through local delivery. The fourth consideration is the advantage of standardization such as drug precise control of dose and volume. The fifth consideration, although remains at its preliminary stage and requires further validation, is the ability to augment the performance of acupuncture (namely “synergy”). For the last point, however, we do have intensive data generation in KOA mice that received daily lidocaine‐laden *nd*‐Acu treatment for 28 days. According to the Evidence‐Based Clinical Practice Guideline of KOA, we decided to use lidocaine as the first API since it is suitable for intra‐articular osteoarthritis treatment in clinical and fits the structure requirement of inclusion complex design.^[^ ^12^ ^]^ Our results showed that preclinical efficacy enhancement was supported by biochemical assays, histology assessment, behavior observation, and proteomics data which will be discussed below.

## 2 Results and Discussion

### 2.1 Combined Use of Electrochemistry and Inclusion Complex Design to Develop *nd*‐Acu

**Figure 1:**
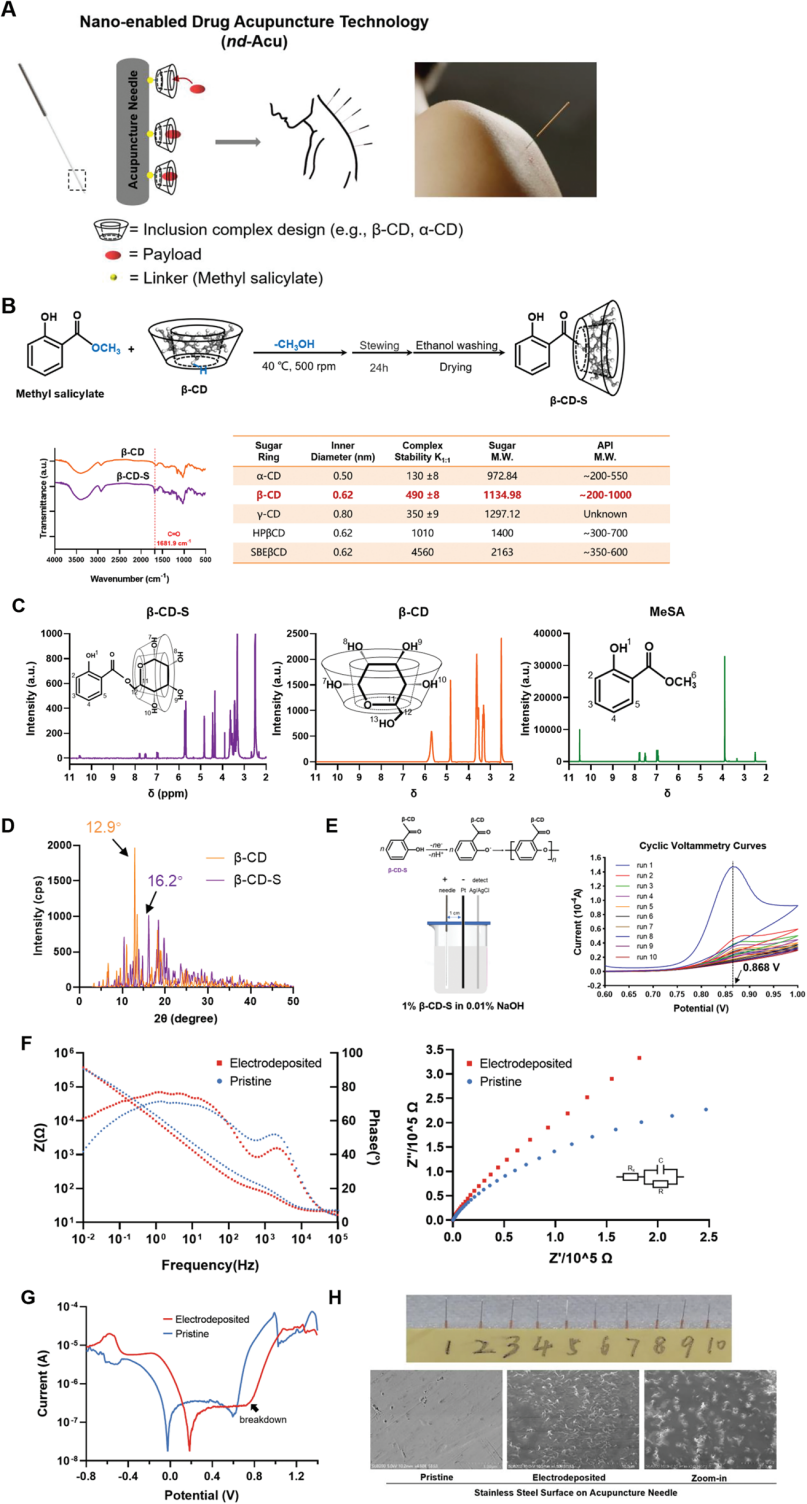
Combined use of electrochemistry and inclusion complex designs to develop *nd*‐Acu. A) Schematic of *nd*‐Acu design. The essential design feature is the covalent attachment of β‐CD derivative to the stainless surface where inclusion complexation mediated drug encapsulation occurs. This endows the classic acupuncture needle with a new feature, i.e., drug encapsulation of chemically defined payloads. B) Chemical reactions for the synthesis of β‐CD‐S, the intermediary product that was deposited on the needle surface. FT‐IR spectra of β‐CD (orange) and β‐CD‐S (purple). Inclusion complexation is sensitive to the chemical structures of payload molecules and host molecules, as we outlined in the inserted table. C) ^1^H NMR (400 MHz, DMSO‐d_6_, δ) spectrum of β‐CD‐S (purple), β‐CD (orange), and MeSA (green) in DMSO‐d_6_. D) Powder X‐ray diffraction patterns of β‐CD (orange) and β‐CD‐S (purple). E) Scheme of electrodeposition reaction and cyclic voltammetry curves of a representative acupuncture needle in 1% β‐CD‐S in 0.01% NaOH at a scanning rate of 5 mV s^−1^ and ambient temperature for ten runs. F) Bode (left) and Nyquist (right) plots of pristine (blue) and electrodeposited (red) acupuncture needles. G) Tafel plots of pristine (blue) and electrodeposited (red) acupuncture needles in 0.01 mol L^−1^ PBS. H) Photograph of electrodeposited acupuncture needles. Ultrastructural visualization of pristine and electrodeposited acupuncture needle using SEM.

Different from physical absorption, a key design feature for *nd*‐Acu is to use the inclusion of complex structure for drug encapsulation. This required the chemical modification of the host molecules, which were subsequently attached to the stainless‐steel surface via an electrochemical process (**Figure** **1A**). The hydrophobic inner cavity of the host molecule such as cyclodextrins provides the possibility to encapsulate payload molecules. As shown in the inserted Table in Figure 1B,^[^ ^13^ ^]^ a wide range of cyclodextrin molecules of distinct inner space and sugar ring size are applicable to this design, allowing the loading of various payloads with different physicochemical properties. For proof‐of‐principle, we used β‐Cyclodextrin (β‐CD), an FDA‐approved drug excipient, as the host molecule for further investigation.

### 2.2 Synthesis and Characterization of β‐CD‐S

Although β‐CD per se was inefficient for the needle attachment due to the inefficient binding affinity, methyl salicylate (MeSA) was used as a “linker” to connect the β‐CD and acupuncture needles. This enabled phenol and phenolic derivatives to form an insulating film coating on the surface of stainless steel under electropolymerization. Specifically, MeSA and β‐CD undergo an esterification reaction at 40 °C to form β‐cyclodextrin salicylate (β‐CD‐S) (Figure 1B). This important intermediate product was characterized by FTIR (Figure 1B), ^1^H NMR (Figure 1C), and XRD (Figure 1D), before use for drug loading. While the detailed interpretation was available online, the most recognizable FTIR signature was the peak at 1681.9 cm^−1^ in β‐CD‐S (the purple line in Figure 1B), which was absent in the pure β‐CD group (the orange line in Figure 1B). This demonstrated the presence of aromatic carbonyl (C═O),^[^ ^14^ ^]^ confirming that the methyl salicylate was covalently linked to β‐CD. In the ^1^H NMR analysis, the fingerprint of β‐CD‐S included *δ* = 7.0, 7.5, 7.8, and 10.5 ppm, which demonstrated the existence of MeSA in β‐CD‐S. Through integral analysis, this chemical shift can also be used to determine the binding rate of MeSA binding in β‐CD, i.e., ≈0.46:1 (molar ratio), similar to the MeSA binding efficiency (BE) calculated in Table S1 (Supporting Information).^[^ ^15^ ^]^ The major chemical shift of β‐CD and methyl salicylate did not change significantly after forming β‐CD‐S, indicating that MeSA was attached to the surface of β‐CD rather than being trapped inside the β‐CD cavity.^[^ ^16^ ^]^ Furthermore, the XRD patterns of β‐CD versus β‐CD‐S demonstrated the shift of crystal structure after MeSA binding (Figure 1D). Impressively, the XRD intensity has decreased in β‐CD‐S, indicating the reduced degree of crystallinity. Similar to the literature, the characteristic peak of β‐CD (at 12.9°) disappeared in the β‐CD‐S sample and a new peak emerged at 16.2°, which further validated the formation of β‐CD‐S.^[^ ^17^ ^]^

### 2.3 Electrodeposition of β‐CD‐S on the Surface of Acupuncture Needles

The resulting β‐CD‐S compound was electrodeposited on the surface of acupuncture needles using an electrochemical workstation in a three‐electrode system. In 0.01% NaOH solution, the phenol hydroxyl group of β‐CD‐S was reduced in the working electrode by losing an H^+^ proton, accompanied by the formation of phenoxy radicals and their intermediates on the surface of the needles. This led to the deposition of poly (phenoxy) film, in an irreversible fashion, which endowed the *nd*‐Acu with the ability to load payload(s) of interest (Figure 1E, left panel).^[^ ^18^ ^]^ The cyclic voltammetry curves revealed the degree of electrodeposition in a cycle number‐sensitive fashion; the electrodeposition was largely completed from cycle #1 to #5. Owing to the insulator nature of β‐CD‐S, we concluded the β‐CD‐S modification when the cycle number reached ten because the detected current was closed to 0 A (*a.k.a*. surface saturation). Noteworthy, there was a peak in curves at 0.868 V (Figure 1E, right), with a current of 1.47 × 10^−4^ A, which demonstrated that β‐CD‐S was attached to the surface of the needle via electrooxidation at this potential. This process was irreversible because there was no discernable peak when applying reversed current. To further confirm the presence of electrochemically deposited β‐CD‐S on the surface of the needle, in situ FTIR spectrum of the electrodeposited needles sample was characterized. We found a peak at 1681.9 cm^−1^ in electrodeposited needles (the green line in Figure S1, Supporting Information), which also appeared in β‐CD‐S, indicating the presence of aromatic carbonyl (C═O).

The electrodeposition endowed the needles with strong corrosion resistance and biocompatibility. The electrochemical performance was characterized by linear sweep voltammetry (LSV) curves, electrochemical impedance spectroscopy (EIS), and Tafel plots. LSV curves showed that the electrodeposition process only occurred as the β‐CD‐S in the electrolyte, as evidenced by a clear peak demonstrating the procedure of electrooxidation (Figure S2, Supporting Information). EIS confirmed the electrodeposition of β‐CD‐S film and revealed the mechanism underlying the needles' corrosion protection ability (Figure 1F). Specifically, in the low‐frequency region, especially at 0.01 Hz, the electrodeposited needles exhibited a wider phase angle and larger Z modulus, indicating that they had better corrosion protection ability than non‐electrodeposited needles.^[^ ^19^ ^]^ The equivalent circuit of the needles was proposed in the form of a series connection of parallel RC circuits.^[^ ^18^ ^]^ The charge‐transfer resistance was calculated from the Nyquist plots, shown in Table S2 (Supporting Information). The resistance of the electrodeposited needles was 3.476 × 10^5^ Ω cm^−2^, higher than that of pristine needles (1.871×10^5^ Ω cm^−2^). The linear polarization resistance calculated from the Tafel plot (Figure 1G) was increased from 2.9 × 10^6^ to 4.3 × 10^6^ Ω (Figure S3, Supporting Information). Additionally, the breakdown potential of the electrodeposited needles increased to 0.75 V, which is higher than the major redox conditions found in biological systems. This suggested that the *nd*‐Acu needles may have better biosafety compared to other needles.^[^ ^19^ ^]^

Before the biological experimentation, we also looked at the surface morphology of acupuncture needles with or without β‐CD‐S deposition (Figure 1H; Figure S4, Supporting Information). Under optical microscopy, we observed a thin film of ≈5 µm thickness on the surface of the needles after β‐CD‐S attachment, while the surface of pristine needles was relatively smooth (Figure S4, Supporting Information). SEM visualization of the needles’ surface was shown in Figure 1H. Compared to the smooth surface of non‐modified needles, needles treated with β‐CD‐S deposition exhibited “bump”‐like structures on their surface, whereas no such structures were observed on the pristine stainless‐steel surface.

### 2.4 Payload Loading and Release Analyses under Abiotic Conditions and at Intact Animal Level

**Figure 2:**
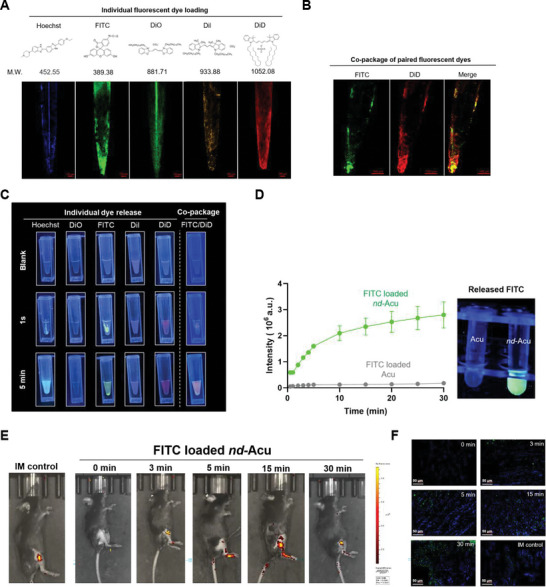
Determination of the characteristics of payload loading and release in *nd*‐Acu. A) Various fluorescent dyes were individually loaded onto β‐CD‐S modified *nd*‐Acu. Fluorescent images of *nd*‐Acu with indicated dyes (scale bar: 100 µm). B) Demonstration of the co‐package of paired dyes (FITC plus DiD) onto *nd*‐Acu (scale bar: 100 µm). C) Images of dye release under UV lighting (365 nm). D) FITC release curves. Left: displayed in fluorescence intensity, *E*_x_: 495 nm, *E*_m_: 525 nm. FITC release from *nd*‐Acu needles (green) and pristine needles (gray) in 0.01 mol L^−1^ PBS at room temperature (*n* = 2). Snapshot of fluorescence image of FITC release at 5 mins under UV (right, 365 nm). E) IVIS images of C57BL/six mice. Mice were under electroacupuncture treatment with FITC *nd*‐Acu for 0, 3, 5, 15, and 30 min. An intramuscular injection of 20 µL at 1 mg mL^−1^ FITC (left) was used as a control. F) Fluorescence images of tissue frozen slices (blue: nucleus, green: FITC fluorescence dye). Tissues were immediately obtained from the experiment (E) after IVIS imaging (scale bar: 50 µm).

Five fluorophores and their combinations were chosen for drug loading and release testing, namely, Hoechst 33 324, Fluorescein Isothiocyanate (FITC), DiOC_18_(3) (Dio), DilC_18_(3) (Dil), and DiIC_18_(5) (DiD). After chemical structure analysis, we deliberately selected these fluorescent dyes because they could fit into the packaging space of β‐CD, as we outlined in **Figure** **2A**. Unloaded *nd*‐Acu needles were incubated with indicated dye solutions, followed by drying steps (Figure 2A). Moreover, we demonstrated the possibility of drug loading using more than one dye, such as the FITC (green color) plus DiD (red color) combination. When co‐packaged FITC and DiD, imaging of green and red channels demonstrated >30% colocalization (yellow color in the merged picture) (Figure 2B). This prompted us to contemplate single or paired drug delivery using the *nd*‐Acu platform, which the latter could be used to achieve drug synergy, a popular concept in medicine including traditional Chinese medicine. To obtain preliminary evidence of dye release, ethanol was chosen to exaggerate the release process of these dyes, which could be captured by a phone camera under UV. Specially, a camera was used to investigate the drug loading and release profile of dye‐laden *nd*‐Acu needles under UV. The investigator could immediately see the released dye in the test tube, 1 s post‐incubation in ethanol solution at room temperature (Figure 2C). The dyes could freely be diffused in the test tube 5 min after incubation. In the case of *nd*‐Acu with co‐packaged FITC/DiD dyes, we saw orange color after incubation, putatively due to the distinct emission wavelengths of each dye. We also detected the dye release under brightfield; similar results were obtained (Figure S5, Supporting Information).

To simulate the drug release in the biological system, 1X phosphate‐buffered saline (PBS, pH 7.2–7.4) was chosen as the release medium for further investigation, instead of ethanol. To confirm the inclusion complex mediated dye loading, we also determined the physical absorption of dye on pristine needles. For quantitative analysis, we repeated the abiotic release experiment using FITC‐laden *nd*‐Acu needles (*n* = 2), followed by fluorescent intensity analysis using a microplate reader. This allowed us to demonstrate a time‐dependent dye release profile, i.e., fluorescent intensity reached a plateau ≈30 min post incubation (Figure 2D). In this case, we have included a control group for physical absorption approach, i.e., FITC attached on non‐modified needle. We were able to demonstrate a 15‐fold more abundant FITC loading and release for *nd*‐Acu than the physical absorption control.

We were encouraged by the promising abiotic drug release data and decided to investigate the dye release at the intact animal level (Figure 2E). C57BL/six mice received acupuncture using FITC‐laden *nd*‐Acu. The acupuncture points, GB‐34 (Yanglingquan) and ST‐36 (Zusanli) were determined by a certified specialist. Under anesthesia, FITC loaded *nd*‐Acu remained in the animal tissue for 0–30 min, followed by IVIS imaging. We also conducted intramuscular injection of the identical amount of FITC dye for comparison (Figure 2E, the first panel). Initial reference images were obtained before the use of FITC‐laden *nd*‐Acu to show a very low background in the mice (Figure 2E, the second panel). The lateral position images indicated a gradual dye release, peaked at ≈15 min post acupuncture (Figure 2E, the third to sixth panels). Interestingly, we indeed discerned an FTIC diffusion profile using FITC‐laden *nd*‐Acu. At the conclusion stage of the in vivo release experiment, animal biopsies were collected to prepare tissue slices for fluorescent detection (Figure 2F). Consistent with the whole animal image, the fluorescent signal visualization was in agreement with the IVIS data.

### 2.5 Proof‐of‐Principle Efficacy Study Using Lidocaine‐Laden *nd*‐Acu to Treat MIA‐Induced KOA in Mice

While we have demonstrated the possibility of dye loading/release using *nd*‐Acu, it is essential to show the therapeutic efficacy of using an active pharmaceutical ingredient or API‐laden *nd*‐Acu in a disease model. For comparison purposes, it is also important to include necessary controls such as clinical standard care and acupuncture alone in the efficacy study. For proof of principle, we decided to load lidocaine, a local anesthetic, on *nd*‐Acu to treat knee osteoarthritis (KOA). The reasons behind this decision include that 1) the chemical structure of lidocaine meets the requirement for *nd*‐Acu drug encapsulation, 2) classic acupuncture is regarded as an alternative treatment for KOA with proven benefits in human and KOA mouse models,^[^ ^20^ ^]^ and 3) in‐depth understanding of the pathophysiology of KOA process at the molecular level (**Figure** **3A**).^[^ ^21^ ^]^ More importantly, there is clinical evidence to suggest that combining acupuncture with local injections of various drugs (e.g., lidocaine and hyaluronic acid)^[^ ^22^ ^]^ was more effective than acupuncture alone; the lidocaine patient study involved a decent patient number, i.e., 180 patients.^[^ ^23^ ^]^

Figure 3Proof‐of‐principle efficacy study using lidocaine‐laden *nd*‐Acu to treat MIA‐induced KOA in mice. A) Schematic diagram of working mechanisms by lidocaine based on literature information. The favorable chemical structure of lidocaine prompts us to consider *nd*‐Acu for drug encapsulation. B) Determination of lidocaine release from *nd*‐Acu needles in 0.01 mol L^−1^ PBS at room temperature evaluated by HPLC. Lidocaine release from pristine acupuncture needles was included as a control. C) Schematic illustration of electroacupuncture treatment by lidocaine‐laden *nd*‐Acu to MIA‐induced KOA mice (top). Inserted box: Photos of electro‐acupuncture equipment, animal holders, and procedure parameters (bottom left) and location of acupoint GB‐34 (Yanglingquan) and ST‐36(Zusanli). Post MIA injection, the mice received daily treatment using lidocaine *nd*‐Acu for 28 days, followed by animal sacrifice at day 32. The controls included non‐treated KOA mice (NT), electro‐acupuncture treatment by classic needles, lidocaine acupoint injection, and TAA articular injection. We also included healthy mice as control. D) At the conclusion stage of the efficacy study, IL‐1β, IL‐6, and TNF‐α levels in knee articular cavity fluid were determined by ELISA (*n* = 6). E) Representative histological assessment for cartilage damage by Safranin‐O staining. A total of 18 randomly selected knee joint samples were used for this analysis, followed by the semi‐quantitative evaluation for the OARSI score (right, *n* = 3). F) Synovial inflammation assessed by H&E staining (left). The quantitative analysis of the synovitis score was provided on the right panel (*n* = 3). G) Representative microcomputed tomography (µCT) images of mice in the indicated groups. (*p < 0.05; ^**^ *p* < 0.01; ^***^ *p* < 0.001; ns = non‐significant.).

**Figure:**
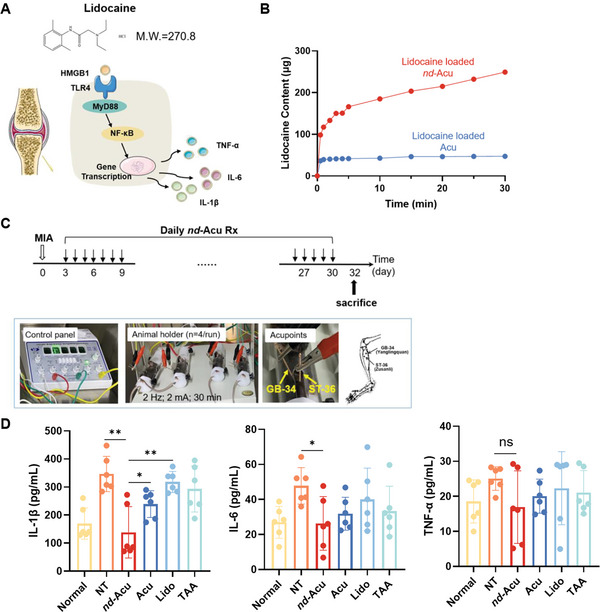

**Figure:**
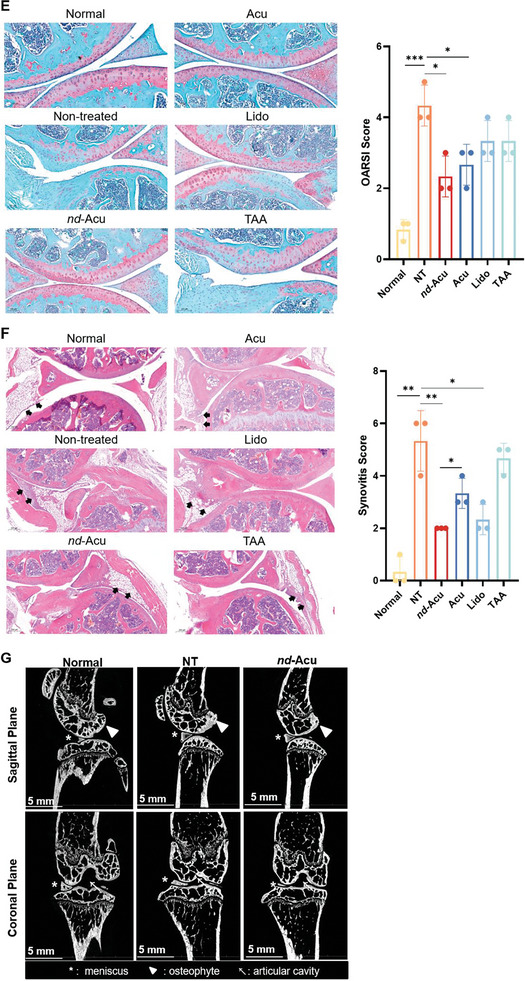

Against this background, we hypothesized that lidocaine *nd*‐Acu could be used to effectively treat KOA in mice. To test this hypothesis, we used a similar protocol to load lidocaine, followed by the quantitative drug release experiment using HPLC. In agreement with the dye release experiment, we observed a time‐dependent lidocaine release from 0 to 30 min post‐incubation in 1 × PBS solution at room temperature. The drug release profile is neglectable using pristine needles of physically attached API (Figure 3B). Based on the abiotic release experiment, we estimated that a single *nd*‐Acu needle could deliver ≈50 µg lidocaine in vivo after acupuncture for 30 min. To investigate the effectiveness of lidocaine‐laden *nd*‐Acu, we established a KOA mouse model induced by monosodium iodoacetate (MIA) based on a published protocol.^[^ ^20^, ^24^ ^]^ Three days after MIA injection, the mice received daily electro‐acupuncture using lidocaine‐laden *nd*‐Acu for a duration of 28 days. While the detailed experimental setup and acupuncture procedure (by a certified specialist) were described in the method section, it is sufficed to mention here that the acupuncture points were GB‐34 (Yanglingquan) and ST‐36 (Zusanli), a popular choice for acupuncture experimental research in KOA rodents (Figure 3C, inserted box).^[^ ^25^ ^]^ We have included the necessary controls to the maximum extent possible in a single mouse experiment, such as normal mice, non‐treated KOA mice, acupuncture alone without drug, and lidocaine acupoint injection. In the same experiment, we also included the standard care treatment using triamcinolone acetonide (TAA) injection in the articular cavity. After *nd*‐Acu treatment for one month, the animals were sacrificed on day 32.

### 2.6 Post‐Sacrifice Analyses in the Efficacy Study Using Lidocaine *nd*‐Acu: Cytokine Quantification, Histology Examination, and High‐Resolution CT Scan

While we will discuss the qualitative endpoints later (e.g., behavior test and pain assessment), in the first efficacy experiment, we deliberately selected objective and quantitative parameters such as pro‐inflammatory cytokines and histology analysis. Impressively, the treatment using lidocaine *nd*‐Acu significantly reduced the levels of IL‐1β in the articular cavity fluid, which were extracted from the supernatant of the homogenated knee joint, outperforming all the controls (Figure 3D, left panel). We also observed a reduction of IL‐6 level for lidocaine *nd*‐Acu compared to non‐treated control (Figure 3D, middle panel). In the case of TNF‐α, although we saw a trend of decreased cytokine release, the change was not statistically significant (Figure 3D, right panel).

To further evaluate the effects of lidocaine‐laden *nd*‐Acu, histological analysis was performed to examine cartilage damage and synovial inflammation, using Safranin‐O staining and H&E staining, followed by Osteoarthritis Research Society International (OARSI) score and synovitis score analysis respectively (Figure 3E,F; Figure S6, Supporting Information).^[^ ^26^ ^]^ In mice with KOA, cartilage proteoglycan depletion can be visualized through Safranin‐O staining, which highlights uneven coloring in the femoral plateau and tibial plateau quadrants of the joint, as compared to normal mice (Figure 3E). Overall, electro‐acupuncture treatment was found to be beneficial, particularly when using a lidocaine‐laden *nd*‐Acu, as it effectively corrected the depletion of cartilage proteoglycan. The OARSI analyses conducted by two independent researchers showed that electro‐acupuncture treatment with lidocaine‐laden *nd*‐Acu significantly reduced the OARSI score (*p = 0.0132, lidocaine *nd*‐Acu vs non‐treated; *p = 0.0241, electro‐acupuncture vs non‐treated), followed by electro‐acupuncture with pristine needles. Acupoint injection with lidocaine was also effective in reducing the OARSI score. Validation of Safranin‐O staining was carried out using H&E staining and synovitis score analysis (Figure 3F; Figure S6, Supporting Information). In normal mice, the synovium surface was found to be smooth, intact, and had a single layer of lining cells, with normal cellularity of the synovial stroma. In contrast, KOA mice exhibited a significant increase in the number of lining cells and stromal cells in the synovium surface, indicating the presence of synovitis in the joint. Electro‐acupuncture, especially using a lidocaine‐laden *nd*‐Acu, was found to be effective in reducing synovitis (*p = 0.016, lidocaine *nd*‐Acu vs acupuncture). Synovitis score analysis showed that lidocaine‐laden *nd*‐Acu gave the strongest score reduction, and was consistent with the degree of cartilage damage.

In light of the promising efficacy results using lidocaine‐laden *nd*‐Acu, we also performed micro‐computed tomography (µ‐CT) scan, using mice from a repeat experiment. Due to the logistic reason, we limited the treatments to normal, non‐treated, and lidocaine‐laden *nd*‐Acu groups for the scan and proteomic study. The static pictures cut from 3D videos in Figure 3G showed the sagittal and coronal orientation of the knee joint. In the sagittal plane orientation, it was observed that KOA mice developed subchondral bone sclerosis and osteophytes at the femoral condyle (Figure 3G, top and middle panels). Lidocaine‐laden *nd*‐Acu was found to alleviate osteophyte formation (Figure 3G, top‐right panel), while normal mice showed no osteophytes (Figure 3G, top‐left panel). Additionally, in the coronal orientation, KOA led to a decreased joint spacing in the articular cavity (white arrows in Figure 3G, bottom and middle panels), which was prevented by lidocaine‐laden *nd*‐Acu. This suggested that lidocaine‐laden *nd*‐Acu can prevent KOA development by reducing bone structural damage.

### 2.7 Mechanistic Understanding for Lidocaine‐Laden *nd*‐Acu in Treating KOA

We were encouraged by the in vivo efficacy of lidocaine *nd*‐Acu, and the key problem became how it works mechanistically. While it could be too early to summarize a definitive blueprint of the working mechanism for *nd*‐Acu, we decided to use combined approaches namely hypothesis‐driven experiment and non‐biased proteomic research to answer this mechanistic question.

For the former approach, we were inspired by the previous studies, which demonstrated electro‐acupuncture may exert its anti‐inflammatory effects by suppressing the HMGB1‐TLR4‐NF‐κB signaling pathway.^[^ ^27^ ^]^ Interestingly, in addition to the anesthetic effects, lidocaine might also impact HMGB1 and TLR4 pathways.^[^ ^28^ ^]^ For example, it was reported that lidocaine can inhibit the release of HMGB1 from THP1 macrophages. Will our lidocaine *nd*‐Acu inhibit these pathways? We retrieved the animal samples from the first efficacy experiment described in Figure 3, followed by a qPCR experiment. While acupuncture alone or lidocaine exerted more or less inhibitory effect on the mRNA levels of HMGB1 and TLR4, lidocaine *nd*‐Acu gave the strongest inhibitory outcome, a potential mechanism to interpret the best‐performing efficacy in the mouse data (**Figure** **4A**).

Figure 4Mechanistic understanding of lidocaine‐laden *nd*‐Acu in treating KOA. A) Normalized levels of HMGB1 and TLR4 mRNA in different treatment groups (*n* = 5). The samples came from the first efficacy study as we discussed in Figure 3. B) In a repeated efficacy study, KOA mice received lidocaine‐laden *nd*‐Acu, similar to Figure 3. Time‐dependent analysis of thermal withdrawal threshold in the normal mice versus non‐treated versus lidocaine‐laden *nd*‐Acu treated KOA mice (left panel, *n* = 8). Cartilage damages were assessed by Safranin‐O staining (right panel). These essential endpoints confirmed our discovery in Figure 3 and ensure the sample quality for the proteomics experiment. C) Hierarchical cluster analysis heatmap of partial differentially expressed proteins among healthy mice, non‐treated KOA mice, and *nd*‐Acu treated KOA mice. This reveals the potential role of small GTPase proteins, such as cdc42 (*n* = 3). D) Use of immunoblotting to confirm the proteomics data for the level of CDC42 protein in healthy mice, non‐treated KOA mice, and *nd*‐Acu treated KOA mice (*n* = 3). E) The results in (D) behooved us to also revisit the tissue blocks in the first efficacy study in which we were able to show reduced cdc42 after lidocaine‐laden *nd*‐Acu treatment through IHC staining. Double‐headed arrow: cartilage region; brown color: cdc42 positive. F) Bubble diagram of the Gene Ontology (GO) enrichment analysis. This analysis reveals the potential role of mitochondria‐related pathways during the treatment by lidocaine‐laden *nd*‐Acu. G) Bubble diagram of the Kyoto Encyclopedia of Genes and Genomes (KEGG) enrichment analysis based on DEPs analysis. The top 10 KEGG pathways were shown according to the *p‐value* of the rich factor. (*p < 0.05; ^**^ *p* < 0.01; ^***^ *p* < 0.001, ^****^ *p* < 0.0001.).

**Figure:**
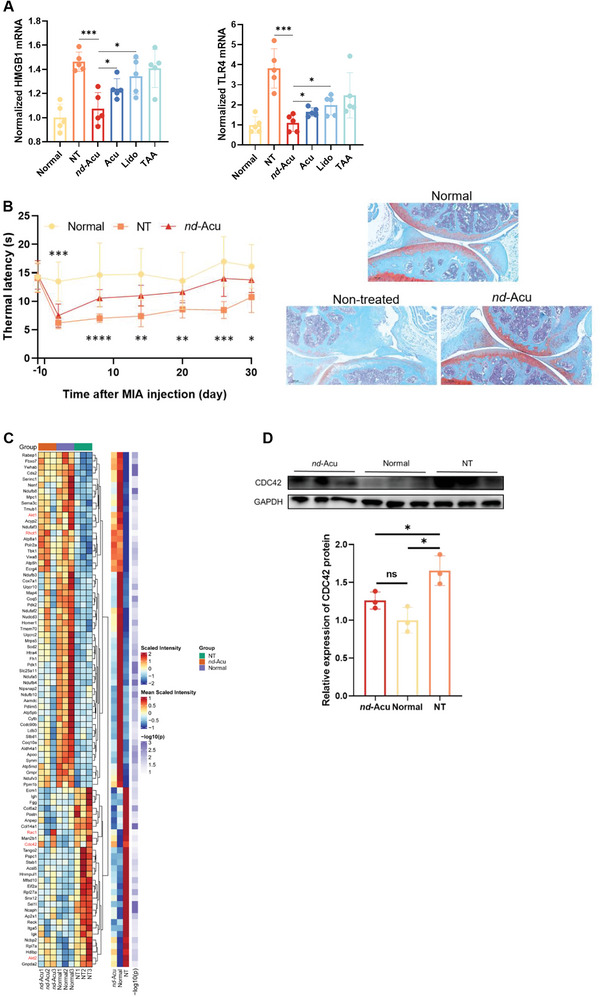

**Figure:**
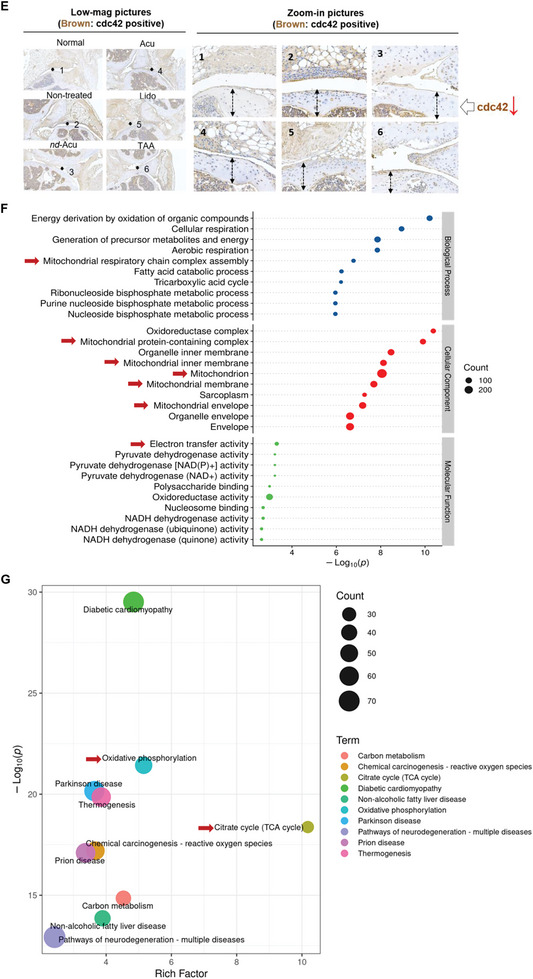

For the proteomic approach, we repeated the essential components in the efficacy study to ensure the best sample quality for the expensive OMICS research. We limited our group design to normal mice, non‐treated KOA mice, and lidocaine *nd*‐Acu‐treated mice. The treatment dose and schedule were similar to Figure 3. The in vivo effectiveness of lidocaine *nd*‐Acu was first confirmed by the Safranin‐O staining and pain hypersensitivity study. After one week of the treatment with lidocaine‐laden *nd*‐Acu, the mice showed a significant increase in thermal withdrawal latency. This trend persisted throughout the entire experiment as shown in Figure 4B. Moreover, lidocaine *nd*‐Acu alleviated the severity of KOA, evidenced by the Safranin‐O staining assay. After the confirmatory efficacy study, we used the cartilage cell samples that were harvested from the knee joints for the proteomic analysis (Figure 4C **)**.

Overall, our proteomic study has identified over one thousand differential proteins that were either up‐regulated or down‐regulated; and the most prominent hits were visualized by a heatmap display (Figure 4C; Figure S7, Supporting Information). The beauty of proteomic data is the identification of molecular targets via a non‐biased approach, which is particularly suitable for the mechanistic study of *nd*‐Acu. In the differentially expressed proteins (DEPs) analysis, a list of interesting proteins emerged (Figure S8, Supporting Information), such as cdc42, rac1, and akt2, which prompted us to consult the literature.

There is published evidence to suggest that decreased level of cdc42, a small GTPase protein, could be beneficial for KOA management.^[^ ^29^ ^]^ In the context of osteoarthritis, cdc42 has been shown to regulate chondrocyte differentiation and matrix synthesis, which are important processes for maintaining cartilage homeostasis. A previous study found that small GTPases, including cdc42, accelerate the rate of chondrocyte differentiation and apoptosis, suggesting the essential roles of these GTPases in chondrocyte hypertrophy.^[^ ^30^ ^]^ Preliminary studies have also illustrated that Rac1 inhibition suppressed OA articular chondrocytes from undergoing hypertrophy‐like changes both in vivo and in vitro.^[^ ^29^ ^]^ Based on the abovementioned awareness, we performed an immunoblotting assay to evaluate cdc42 protein expression. In agreement with the proteomic data, we saw a reduced level of cdc42 after lidocaine *nd*‐Acu treatment. We also retrieve the tissue blocks in the first efficacy, which further confirmed the blotting results (Figure 4D,E). Collectively, we believe that GTPases‐related pathways (that were moderated by lidocaine *nd*‐Acu) could positively contribute to the in vivo efficacy. With respect to Akt2, the role of this protein is not well understood in osteoarthritis; we did not have a definitive answer about why Akt2 was changed in the *nd*‐Acu group.

Moreover, inspiring patterns also emerged in the Gene Ontology (GO) enrichment analysis. This analysis revealed that the DEPs were significantly enriched in processes related to the mitochondrion, including mitochondrial respiratory chain complex assembly, mitochondrial protein‐containing complex, mitochondrial inner membrane, mitochondrial membrane, mitochondrial envelope, and electron transfer activity (Figure 4F). Moreover, the Kyoto Encyclopedia of Genes and Genomes (KEGG) enrichment analysis of DEPs further confirmed the key role of mitochondrion since it is involved in citrate cycle (TCA cycle) and oxidative phosphorylation (Figure 4G). Interestingly, previous studies have shown that mitochondrial dysfunction in chondrocytes was associated with increased oxidative stress and inflammation, which are key features of KOA.^[^ ^31^ ^]^ Additionally, impaired mitochondrial function in other cells involved in the joint, such as synovial cells and macrophages, may also contribute to the development and progression of KOA.^[^ ^32^ ^]^ In addition to mitochondria‐related pathways, it also reveals hints in terms of pathways that correlate to cellular respiration and oxidoreductase activity (among others). This prompted us to conduct further mechanistic investigations, which require separate planning in the future. Take the mitochondria pathway for example, the putatively involved biological process could include ROS, SOD, SIRT3, et al. To avoid the overinterpretation of the proteomics data, however, we want to point out that the proteomics data is highly complicated and therefore still requires careful interpretation to precisely understand the mechanism. Nevertheless, we believe that it is important to explore the working mechanisms of *nd*‐Acu, an upgraded version of classic acupuncture that has been sometimes criticized for lack of mechanistic understanding. Our comprehensive nano‐manufacture and biology data experimentally demonstrate that *nd*‐Acu is a versatile nano‐enabled platform that can be used for implementing acupuncture and drug delivery. Our proof‐of‐principle efficacy data also demonstrated that this platform holds great promise for treating various diseases, including KOA.

## 3 Conclusion

In this study, we successfully innovated a nano‐enabled drug delivery acupuncture technology (*nd*‐Acu) to enhance the therapeutic effect in a KOA murine model through complementary and combined acupuncture and drug delivery effects. Our comprehensive nano‐manufacture and biological data, combined with mechanistic exploration (such as TLR4‐NF‐κB and cdc42 pathway), experimentally demonstrate that *nd*‐Acu is a versatile nano‐enabled platform that can be used for implementing acupuncture and drug delivery concurrently. This platform holds great promise for treating various diseases, including KOA.

## 4 Experimental Section

### Materials

β‐Cyclodextrin (β‐CD) (CAS# 7585‐39‐9, Macklin), isopropanol (CAS# 67‐63‐0, Macklin), ethanol (CAS# 64‐17‐5, Macklin) were purchased from Shanghai McLean Biochemical Technology Co., Ltd. Methyl salicylate (MeSA) (CAS# 119‐36‐8, Leyan) was from Shanghai Haohong Biomedical Technology Co., Ltd. Fluorescein Isothiocyanate (FITC) (CAS# 3326‐32‐7, Coolaber), 1,1′‐Dioctadecyl‐3,3,3′,3′‐Tetramethylindodicarbocyanine, 4‐Chlorobenzenesulfonate Salt (DiIC_18_(5), DiD) (CAS# 127274‐91‐3, Coolaber) were obtained from Shenzhen Wenle Biotechnology Co., Ltd. 1,1 ‘‐ octadecyl‐3,3,3′, 3′‐tetramethylcyanamide perchlorate (DilC_18_(3), Dil) (CAS# 41085‐99‐8, Cayman) was purchased Beijing Biotech Co., Ltd. Lidocaine hydrochloride (CAS# 73‐78‐9, Aladdin) and sodium iodoacetate (CAS# 305‐53‐3, Aladdin) were from Shanghai Aladdin Biochemical Technology Co., Ltd. Triamcinolone acetonide (CAS# 76‐25‐5, Solarbio), 3,3′‐ dioctadecyl oxycarbocyanine perchlorate (DiOC_18_(3)) (CAS# 34215‐57‐1, Solarbio), Hoechst 33 342 (CAS# 875756‐97‐1, Solarbio), and Trizol (Invitrogen) were from Beijing Solarbio Technology Co., Ltd. Acupuncture needle (Hwato, 13 mm length, 0.25 mm diameter) was purchased from Suzhou Medical Supplies Factory Co., Ltd. Recombinant Anti‐CDC42 antibody [EPR15620] (ab187643) was obtained from Beijing Chengzhikewei Biotechnology Co., Ltd.

### Mice

Eight‐week‐old female C57BL/six mice were purchased from Sperford (Beijing) Biotechnology Co., Ltd. The animals were maintained at 21–22 °C, humidity (48‐52%), in a conventional 12:12 light/dark cycle, and had free access to food and water. The experimental protocol received ethical approval from the Animal Care and Use Committee at National Center for Nanoscience and Technology (NCNST21‐2209‐0605).

### Synthesis of β‐CD‐S

The synthesis of *nd*‐Acu needles was designed based on literature with minor modifications.^[^ ^18^ ^]^ Briefly, 11.3 g (0.01 mol) β‐CD was added to a 300‐mL beaker containing 33.3 mL ethanol and 66.7 mL distilled water. The suspension was mixed at 60 °C under magnetic stirring (1,000 rpm) for 1 h. Then, the mixture was naturally cooled to 40 °C at room temperature under magnetic stirring (1,000 rpm). Next, 6 g (0.04 mol) of methyl salicylate was added to the suspension. The mixture was stirred at 40 °C for 4 h to form the β‐CD‐S sediment and stood at 4 °C overnight. After that, the sediment was collected via a decompression filtration process and washed twice using ethanol. Finally, the resulting sediment was dried at 50 °C in the oven for 4 h and kept at room temperature.

### Physical and Chemical Characterization of β‐CD‐S

The important intermediary product, β‐CD‐S, was comprehensively characterized using the following methods.

*FT‐IR*: FT‐IR spectrum of β‐CD‐S and β‐CD were detected via a Fourier transform infrared spectrometer (Spectrum One, Perkin Elmer Instruments Co. Ltd.) in a KBr pellet. The FTIR spectrum of the electrodeposited needles sample was characterized in attenuated total reflectance (ATR) mode via a Fourier transform infrared spectrometer (Spectrum One, Perkin Elmer Instruments Co. Ltd.)

*^1^H NMR (400 MHz, DMSO‐d6, δ)*: ^1^H NMR data of β‐CD, methyl salicylate, and β‐CD‐S were obtained from a nuclear magnetic resonance spectrometer (400 MHz, AVANCE III HD 400). Samples were dissolved in DMSO‐d_6_ and measured at 298 K.

*XRD Analysis*: Powder X‐ray diffraction (XRD) analysis was carried out using an X‐ray diffractometer (D/MAX‐TTRIII, Nippon Koichi Co., Ltd) with Cu Kα (*λ* = 1.54 Å). β‐CD‐S and β‐CD powder were scanned in a 2*θ* range from 3° to 50°at 0.02 s^−1^

### Electrochemical Deposition of β‐CD‐S on Acupuncture Needles

300 mg of β‐CD‐S was dissolved in 30 mL 0.01% NaOH aqueous solution to form an electrolyte solution. Acupuncture needles were washed first using 7.4 v/v % HCl aqueous solution and later using deionized water. An acupuncture needle was used as a working electrode, an Ag/AgCl (in saturated KCl) electrode (6 mm diameter, 65 mm length) was worked as a reference electrode, and a Pt wire (0.5 mm diameter, 37 mm length) was used as a counter electrode. The working electrode was 1 cm away from the counter electrode, as outlined in Figure 1E. An electrochemical workstation (CHI660E, Shanghai Chenhua Instrument Co., Ltd) was used in the electrochemistry production. The scanning range was set at 0.6–1.0 mV and the rate was set to 5 mV s^−1^, respectively. The program began from a potential at 0.6 V, applied forward current first, and cycled ten times. The above‐mentioned condition was regarded as one batch, allowing to complete the β‐CD‐S electrodeposition process on 20 acupuncture needles.

### Electrochemical Characterization

All the tests below were performed in a three‐electrode system with CHI660E electrochemical workstation. Briefly, an acupuncture needle was used as a working electrode, an Ag/AgCl (in saturated KCl) electrode was worked as a reference electrode, and a Pt wire was used as a counter electrode. The working electrode was 1 cm away from the counter electrode. Linear sweep voltammetry of the acupuncture needles was measured at a scanning rate of 5 mV s^−1^ in a range of 0–2 V at room temperature. Pristine needles were submerged in 100 mL of 0.01% NaOH solution with three different solutes, namely 1% β‐CD‐S, 1% β‐CD, and only NaOH. Electrochemical impedance spectroscopy (EIS) of the pristine and electrodeposited needles was carried out at the open circuit potential with a sinusoidal perturbation of ±10 mV over the frequency ranges from 100 kHz to 0.01 Hz at room temperature. 0.01 mol L^−1^ PBS solution was used as an electrolyte. Tafel plots of the pristine and electrodeposited needles were obtained at a scanning rate of 1 mV s^−1^ in a range of −0.4 to 1.2 V at room temperature. 0.01 mol L^−1^ PBS solution was used as the electrolyte, similarly.

### Surface Morphology

The surface morphology of pristine and β‐CD‐S electrodeposited acupuncture needles was observed by scanning electron microscope (SEM, SU8220) and optical microscope (DM2000, Leica).

### Visual Demonstration of Fluorescent Dye(s) Loading and Release from nd‐Acu

Five fluorescent dyes, namely, Hoechst 33 342 (MW: 452.55), DiO (MW: 881.71), FITC (MW: 389.38), Dil (MW: 933.88), DiD (MW: 1052.08) were used in this study. Briefly, acupuncture needles with β‐CD‐S deposition were submerged in 200 µL of ethanol at 1 mg mL^−1^. The incubation was conducted in 1.5 mL Eppendorf tubes and placed at room temperature in the dark for 24 h. After that, the needles were dried at room temperature. Needles with different fluorescent dyes were observed by a Single‐photon confocal microscope (Zeiss 710, Germany). To obtain visual evidence of dye release, the dye‐laden *nd*‐Acu needles were soaked in 200 µL ethanol. Snapshot pictures were taken under UV light (365 nm) at indicated time points. A similar experiment was repeated, followed by picture capture using a brightfield camera. For paired dyes co‐package, the needles were submerged in 200 µL dyes ethanol solution containing FITC and DiD at 1 mg mL^−1^. The abiotic release study was similar to the above.

### Quantitative FITC Release In Vitro

FITC was chosen for quantitative analysis in vitro. FITC‐laden needles were submerged in 200 µL 1X PBS solution at room temperature for a duration of 30 s to 30 min. To determine the abundance of released FITC, the fluorescence intensity was measured by a microplate reader (Synergy HTX, Biotek) (Ex: 495 nm, Em: 525 nm).

### FITC Release In Vivo

Eight‐week‐old female C57BL/six mice were used for observing FTIC release in vivo. Before IVIS imaging, mice received electroacupuncture (EA) treatment with FITC‐laden *nd*‐Acu needles for 0, 3, 5, 15, and 30 min. The acupuncture points were “Zusanli” (ST‐36) and “Yanglingquan” (GB‐34) on the right side. EA parameters were 2 mA current, intensity at 2 Hz frequency, controlled by an EA equipment (Changzhou Yingdi Electronic Medical Device Co., Ltd). As a control, mice received an intramuscular injection of 20 µg FITC in 20 µL 1X PBS solution. Pictures were obtained from the IVIS imaging system (IVIS Spectrum, PerkinElmer). To observe the dye distribution in situ, the mice tissues were rapidly collected after IVIS imaging, followed by OCT embedment and frozen section to prepare 20 µm thick slices (CM1950, Leica). The distribution pattern of FITC fluorescence was observed by an inverted fluorescence microscope (DM IL LED, Leica).

### Lidocaine Hydrochloride Release In Vivo

To load *lidocaine*, β‐CD‐S electrodeposited acupuncture needles were submerged in 200 µL lidocaine hydrochloride 1X PBS solution at 30 mg mL^−1^. The incubation was conducted in 1.5 mL Eppendorf tubes and placed at room temperature in the dark for 24 h. After that, the needles were dried at room temperature. The same experiment was also repeated using pristine needles for comparison. For quantitative analysis, lidocaine‐laden needles were submerged in 200 µL 1X PBS solution at room temperature. To quantify the drug release at an indicated time point, a reverse‐phase high‐performance liquid chromatography (HPLC, Shimadzu LC‐20A XR) equipped with a UV detector at 254 nm was used. A C18 chromatographic column (C18‐139‐2, 5 µm, 250 × 4.6 mm) was used, and sodium phosphate buffer/acetonitrile (50:50) was used as a mobile phase with a 1.0 mL min^−1^ flow rate. The injection volume was 20 µL per run.

### Efficacy Study of Lidocaine nd‐Acu in KOA Mouse Model

Keeping the right knee in a bent position, 25 µL MIA saline solution was intra‐articularly injected into the joint space of the right knee at an MIA dose of 20 mg mL^−1^ using a 31 G needle. Three days after the injection, the mice were used in the efficacy study. Two efficacy experiments were performed, as shown in Figures 3 and 4. In the first efficacy experiment, KOA mice received daily lidocaine *nd*‐Acu (*nd*‐Acu group) for 28 days, followed by animal sacrifice at day 32. The controls included non‐treated (NT group), electroacupuncture treatment by normal needles (Acu group), lidocaine acupoint injection (Lido group), and TAA articular injection (TAA group). Healthy mice was also included as an additional control (normal). To minimize the animal movement, the mice were placed on an animal holder as showed in Figure 3C. KOA mice received indicated EA treatment (via *nd*‐Acu or pristine needle) on the right “Zusanli” (ST‐36) and “Yanglingquan” (GB‐34). The EA parameters were 2 mA current intensity and 2 Hz frequency for 30 min (Changzhou Yingdi Electronic Medical Device Co., Ltd). A certified specialist conducted the procedure based on a published laboratory guideline.^[^ ^25a^ ^]^ The Acu treatment involved the use of pristine needles for daily EA. The mice in the Lido group received 50 µL of 1 mg mL^−1^ Lidocaine hydrochloride at acupoint (ST‐36 and GB‐34) once everyday for 4 weeks. TAA treatment referred to the intra‐articular injection of 25 µL of 1 mg mL^−1^ TAA into the right knee joint once every week for 4 weeks. In the second efficacy study, the essential treatments were repeated, similar to the first efficacy experiment. After confirmation of the in vivo efficacy, the biopsies were used for proteomics work as discussed below.

### ELISA Assay

The concentrations of IL‐1β, IL‐6, and TNF‐α in knee joints were measured with ELISA assays. At the end of the efficacy experiment, the mice's right knee joints were harvested and homogenated by a homogenizer (F6/10 Tissuelyser, Jing Xin) on ice. The resulting mixture was centrifuged at 10 000 rpm at 4 °C for 10 min. Finally, 500 µL supernatant was collected for the measurement of IL‐1β, IL‐6, and TNF‐α levels according to the manufacturer's instructions (IL‐1β Mouse ELISA Kit (BMS6002, Invitrogen), IL‐6 Mouse ELISA Kit (KMC0061, Invitrogen), and TNF‐α Mouse ELISA Kit (BMS607‐3, Invitrogen)).

### Histology Assay

Mice's right knee joints were harvested and fixed in 4% paraformaldehyde. The paraffin blocks were sectioned at a thickness of 5 µm and stained with hematoxylin‐eosin (HE) and safranin O/fast green by a histological service company (Solarbio). Cartilage destruction was scored by two observers under blinded conditions using the OARSI scoring system (grades 0–6). Synovitis was determined by hematoxylin‐eosin staining and synovial inflammation was scored (grade 0–6) (i.e., enlargement of the lining cell layer and density of resident cells).^[^ ^26b^ ^]^ Immunohistochemistry (IHC) of paraffin‐embedded tissues from mice was performed by the histological service company (Solarbio). The primary antibody that recognizes Cdc42 was applied at a 1:250 dilution for IHC.

### µCT Analysis

Some of the fixed mice knee joints were used for CT scan. CT scanning was performed using high‐resolution X‐ray micro‐computed tomography (µCT, Xradia 520 Versa, USA). Images were analyzed and reconstructed with Scout v12.0 and Recon v12.0. 3D model visualization software (XM3DViewer v1.2) was also used. A voltage of 80 kV, a resolution of 7.0 µm per pixel, and a current of 87.5 µA were set for the scanner. The knee joints' coronal, and sagittal movies and images were used for analyses.

### qRT‐PCR

First, the total RNA from the mice's knee joint sample was subjected to the *qRT‐PCR* analysis. Four microgram of total RNA was transcribed into cDNA by HiFiScript cDNA Synthesis Kit (CWBIO). The PCR procedure was performed by Qiagen 208 054 QuantiNova SYBR Green PCR Kit (QIAGEN) and CFX Connect Real‐Time PCR (Bio‐Rad). Primers used for this study were: HMGB1 (forward: 5′‐TGCCTCGCGGAGGAAAATC‐3′, reverse: 5′‐AACGAGCCTTGTCAGCCTTT‐3′), TLR4 (forward: 5′‐AGCTTCTCCAATTTTTCAGAACTTC‐3′, reverse: 5′‐TGAGAGGTGGTGTAAGCCATGC‐3′), and GAPDH (forward: 5′‐TCAACGGCACAGTCAAGG‐3′, reverse: 5′‐TGAGCCCTTCCACGATG‐3′). The amplification program was: one cycle at 95 °C for 2 min, 45 cycles for GAPDH, HMGB1, and TLR4 genes at 95 °C for 15 s, 55 °C for 20 s, 72 °C for 20 s, and finally one cycle for 65 °C for 15 s and 95 °C for 5 s.

### Proteomics Assay

In the confirmatory efficacy study described in Figure 4, cartilage cells were extracted for proteomic research by a service company (Shanghai Bioprofile Technology Co., Ltd). After sample preparation and protein digestion, the resulting peptides were used for TMT labeling according to the manufacturer's instructions (Thermo Fisher Scientific).^[^ ^33^ ^]^ Next, the TMT‐labeled peptides mixture was fractionated by high pH reverse phase fractionation and dried for nano LC‐MS/MS analysis which was performed on a Q Exactive mass spectrometer that was coupled to Easy nLC (Thermo Fisher Scientific). The resulting LC‐MS/MS raw files were imported into Proteome Discoverer 2.4 software (version 1.6.0.16) for data interpretation and protein identification against the database. Analyses of bioinformatics data were carried out with Perseus software, Microsoft Excel, and R statistical computing software. Differentially significant expressed proteins were screened with the cutoff of a ratio fold‐change of >1.20 or <0.83 and *p*‐values < 0.05.^[^ ^34^ ^]^ Expression data were grouped by hierarchical clustering according to the protein level. To annotate the sequences, information was extracted from UniProtKB/Swiss‐Prot, Kyoto Encyclopedia of Genes and Genomes (KEGG), and Gene Ontology (GO). After GO and KEGG enrichment analyses, the construction of protein‐protein interaction (PPI) networks was also conducted.^[^ ^35^ ^]^

### Immunoblotting Assay

After proteomics assay, protein samples of cartilage cells on healthy mice, non‐treated KOA mice, and *nd‐*Acu treated KOA mice were used for western blot assay. Briefly, 12% SDS/PAGE gels were used for electrophoresis, and 15 µg denatured protein was added to each well. After that, 0.22 µm PVDF membranes were used to transfer proteins. Then, 5% BSA (in 1X TBST) was used to block membranes for 2 h. Recombinant anti‐CDC42 (Abcam, ab187643, 1:10 000) was used as the primary antibody, and HRP‐linked goat anti‐rabbit IgG H&L (HRP) (Abcam, ab205718, 1:2000), was used as the secondary antibody. SuperSignal West Pico PLUS (Thermo Fisher, A38554) was used to visualize target bands using the ChemiDoc Touch Imaging System (Bio‐Rad). Image J software was used to analyze the images of immunoreactive bands. The density analysis was used to quantify the expression of CDC42 protein relative to GAPDH.^[^ ^36^ ^]^

### Statistical Analysis

Continuous variables are expressed as mean ± SEM and the difference between groups was analyzed by one‐way ANOVA with LSD test for multiple comparisons. At least three independent samples were contained in each group for each statistical analysis. Column statistics were performed on datasets to check for normal distribution. In all tests, *p*‐values of <0.05 were considered statistically significant. The analysis was conducted using the GraphPad Prism 8.0 software.

## Conflict of Interest

The authors declare no conflict of interest.

## Supporting information

Supporting Information

## Data Availability

The data that support the findings of this study are available from the corresponding author upon reasonable request.
