# Supplementary material for: Effective Treatment of Knee Osteoarthritis Using a Nano‐Enabled Drug Acupuncture Technology in Mice
Source: Adv Sci (Weinh). 2023 Aug 9;10(28):2302586. doi: 10.1002/advs.202302586 (PMC10558644; doi:10.1002/advs.202302586)
Supplement: Supplementary file 1 — Supporting Information [file ADVS-10-2302586-s001.pdf]

ADVANCED SCIENCE
Open Access

Supporting Information

for *Adv. Sci.*, DOI 10.1002/advs.202302586

Effective Treatment of Knee Osteoarthritis Using a Nano-Enabled Drug Acupuncture Technology in Mice

Wenjie Xu, Yu Xiao, Minzhi Zhao, Jiahui Zhu, Yu Wang, Wenbin Wang, Peng Wang and Huan Meng**

Supplemental Information

Effective Treatment of Knee Osteoarthritis Using a Nano-enabled Drug Acupuncture Technology in Mice

Wenjie Xu[1,2,#], Yu Xiao[1,3,#], Minzhi Zhao[1], Jiahui Zhu[1, 4], Yu Wang[1, 4], Wenbin Wang[1, 5], Peng Wang[2]*, Huan Meng[1,3]*

[1] CAS Key Laboratory for Biomedical Effects of Nanomaterials and Nanosafety and CAS Center for Excellence in Nanoscience, National Center for Nanoscience and Technology, Beijing 100190, China

[2] Beijing Hospital of Traditional Chinese Medicine, Capital Medical University, Beijing 100010, China

[3] University of Chinese Academy of Sciences, Beijing 100049, China

[4] Chongqing University of Technology, Chongqing 400054, China

[5] The First Affiliated Hospital of Zhengzhou University, Zhengzhou 450052, Henan, China

#: Equal contribution

* Correspondence author: mengh@nanoctr.cn, wangpeng@bjzhongyi.com

Table S1. Calculation of MeSA binding efficiency.

To calculate the binding rate of MeSA and β-CD in β-CD-S complexes, 5 mg of β-CD-S was dissolved in 100 μL, 0.01% NaOH aqueous solution. Next, the absorbance of the solution was monitored at 305 nm by a multimode microplate reader (Synergy HTX, Biotek).

The concentration of MeSA was obtained through the standard curve (see below). The standard curve showed a linear relationship between the concentration of MeSA and its absorbance at 305 nm. According to the calculation, the average concentration of MeSA was 2.36 mg/mL, which indicated 0.236 mg of MeSA in 5 mg β-CD-S samples. Therefore, our analysis showed that the binding rate of MeSA (121 g/mol) and β-CD (1,117 g/mol) was 0.46:1 mole ratio. The results were obtained from the readouts from two batches.

Batch number	#1			#2			Average
OD value	3.28	3.23	3.10	3.40	3.39	3.34	3.29

MeSA concentration (mg/mL)	2.35	2.31	2.22	2.44	2.43	2.39	2.36

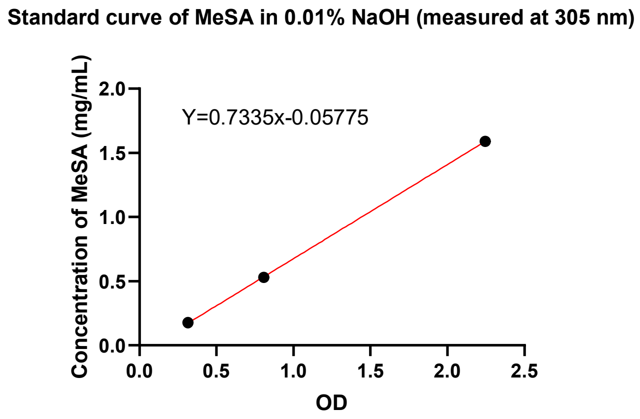

Figure S1. The FTIR spectrum of the electrodeposited needles sample was characterized in attenuated total reflectance (ATR) mode *via* a Fourier transform infrared spectrometer. The same peak at 1681.9 cm^{-1} in electrodeposited needles (the green line in Figure) were found compared to β-CD-S.

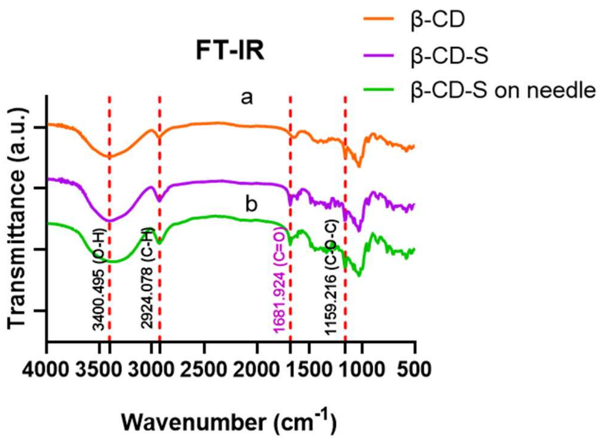

Figure S2. Linear sweep voltammetry curves of pristine acupuncture needles in different electrolytes. The electrolytes: 1% β-CD-S in 0.01% NaOH (orange), 1% β-CD in 0.01% NaOH (purple), and pure 0.01% NaOH aqueous solution (black) (Range: 0-2 V, Scanning rate: 5 mV/s). This data suggested that the electrodeposition process only occurred as the β-CD-S in the electrolyte (but not β-CD), evidenced by the appearance of a clear

peak around 1.0 V (black arrow).

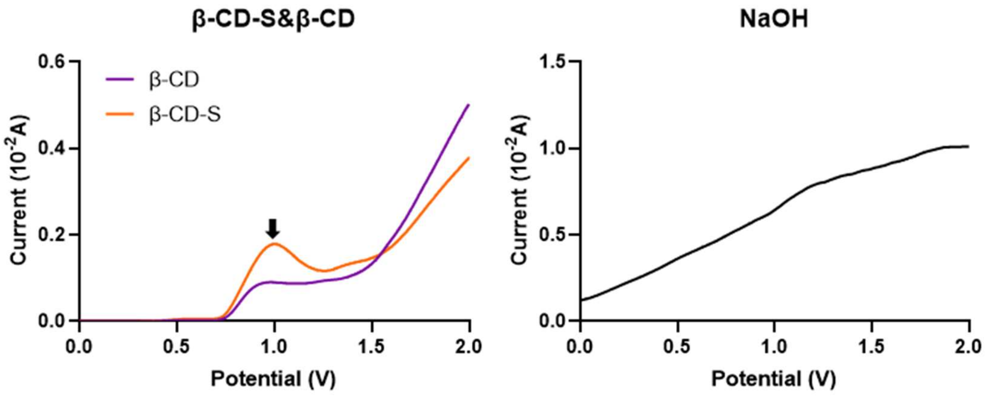

Table S2. Electrochemical parameters obtained from Nyquist plots

Sample	R_s (Ω/cm^2)	R (10^5 Ω/cm^2)	C (10^{-5} F/cm^2)
Electrodeposited needles	28.354	3.476	1.794
Pristine needles	29.624	1.871	1.058

Figure S3. Strong polarization area of Tafel plot (Tafel slope E range: 0.06V), Cathodic (Cat) and anodic (Ano) Tafel curves for electrodeposited needles (blue, top) and pristine needles (red, bottom).

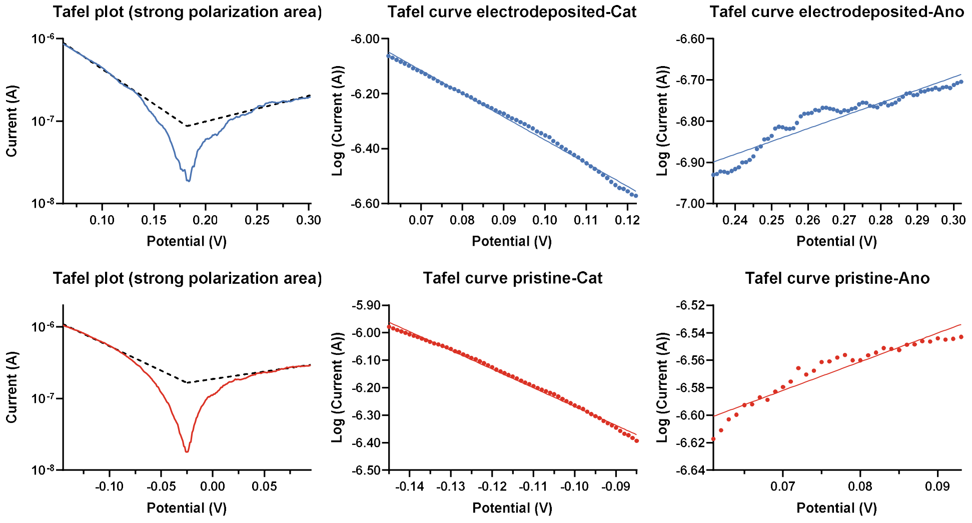

Electrochemical parameters obtained from Tafel plots

Sample	E_{corr} (V)	Cat Slp (1/V)	Cat Int (log i)	Ano Slp (1/V)	Ano Int (log i)	Corr i (10^{-7}A)	Lin Pol R (Ω)
Electrodeposited needles	0.182	-8.445	-5.524	3.121	-7.629	0.87	431522
Pristine needles	-0.025	-6.820	-6.951	2.089	-6.728	1.66	293608

E_{corr}=Corrosion voltage
Cat Slp=Cathodic Tafel slope
Cat Int=Cathodic intercept
Ano Slp=Anodic Tafel Slope
Ano Int=Anodic intercept
Corr i=Corrosion current
Lin Pol R (R_{pol})=Linear polarization resistance
β_{ano}=1/Ano Slp
β_{cat}=-1/Cat Slp
The corrosion voltage and current were obtained from the Tafel curves.

The linear polarization resistance was calculated from the Stern-Geary equation.

Stern-Geary equation $$\mathrm{i}_{\mathrm{corr}} = \frac{\beta_{ano}\beta_{cat}}{2.303R_{pol}(\beta_{ano} + \beta_{cat})}$$

Figure S4. Optical microscope photographs of pristine (bottom) and electrodeposited (top) acupuncture needles (Scale bar: 10 μm). We observed a thin film of ~5 μm thickness on the surface of the needles after β-CD-S electrodeposited (solid arrow). No such finding was observed on the surface of the pristine needles (open arrow).

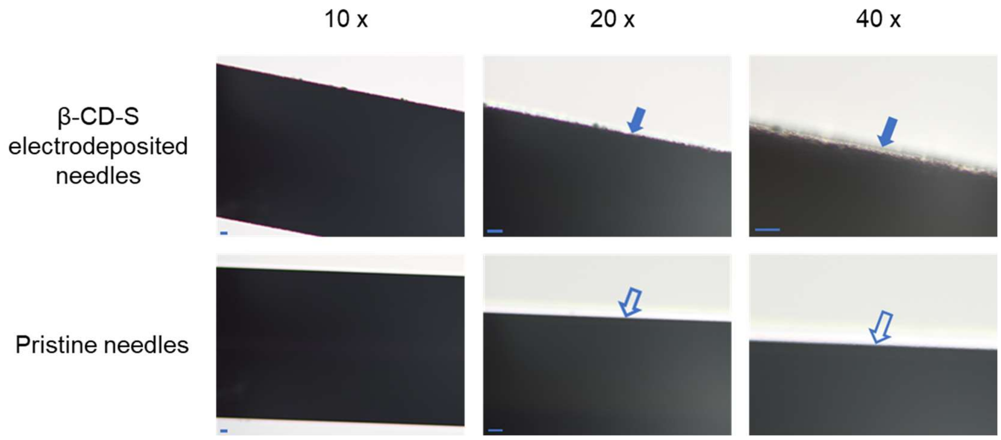

Figure S5. Images of individual and dual dye release in ethanol solution under UV lighting (365 nm) and visible light.

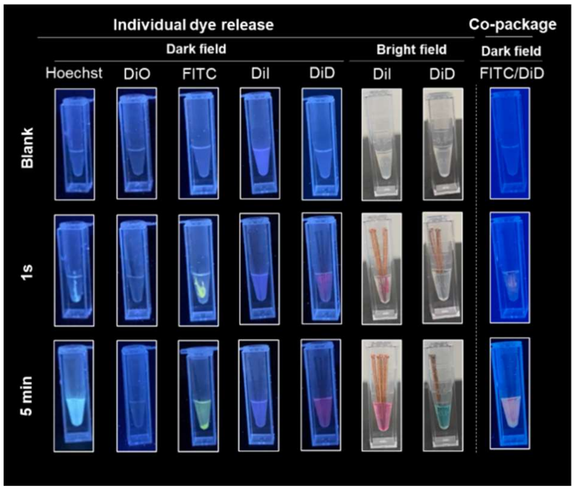

Figure S6. Zoom-in pictures of H&E staining that appears in **Figure 3F**.

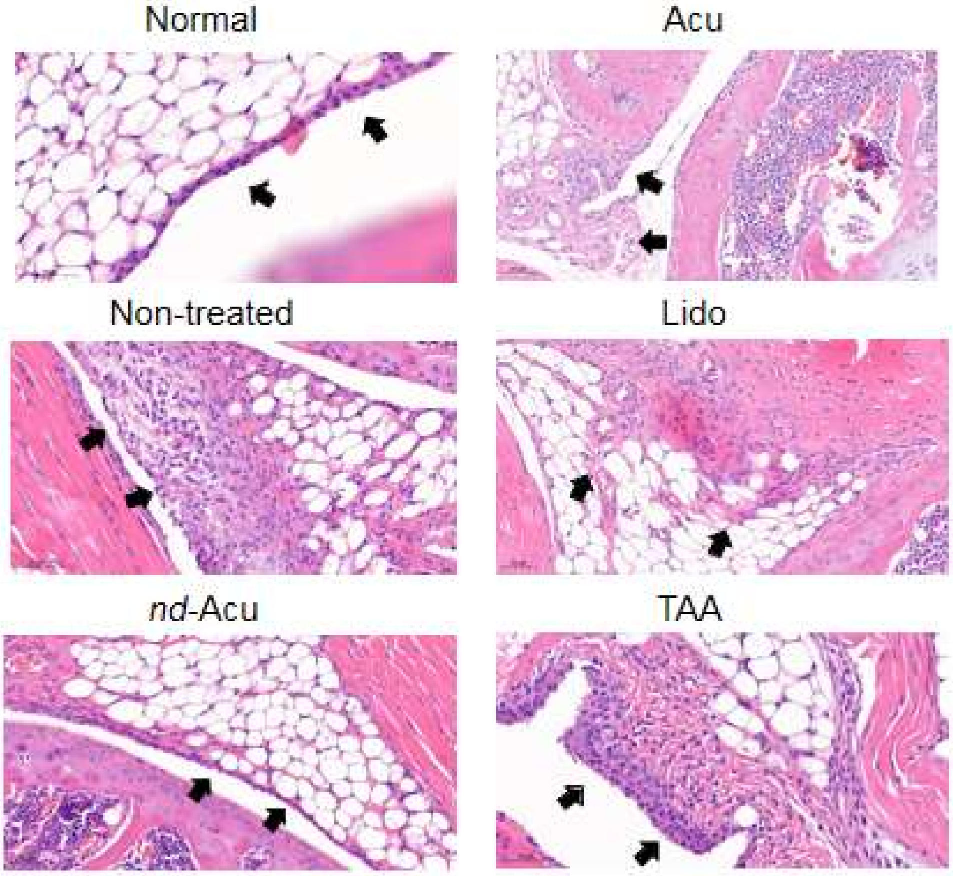

Figure S7. Hierarchical cluster analysis heat map of differentially expressed proteins among normal mice (blue zone on the top), non-treated (pink zone on the top) and *nd*-Acu treatment (green zone on the top). The columns and rows represent the experimental groups and protein names, respectively. Red represents upregulated proteins and blue represents downregulated proteins. Differentially significant expressed proteins were screened with the cutoff of a ratio fold-change of >1.20 or <0.83 and *P*-values < 0.05.

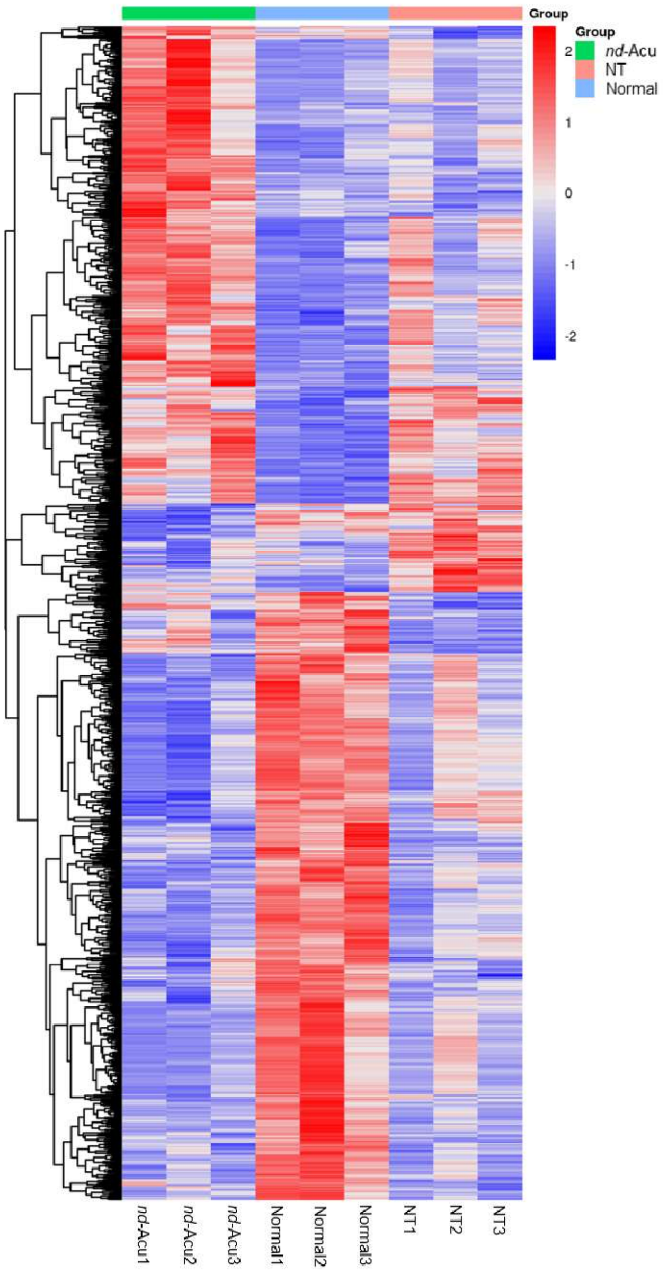

Figure S8. The histogram diagram showed the number of differentially expressed proteins (DEPs) from 3 different groups. Red represents upregulated proteins and blue represents downregulated proteins.

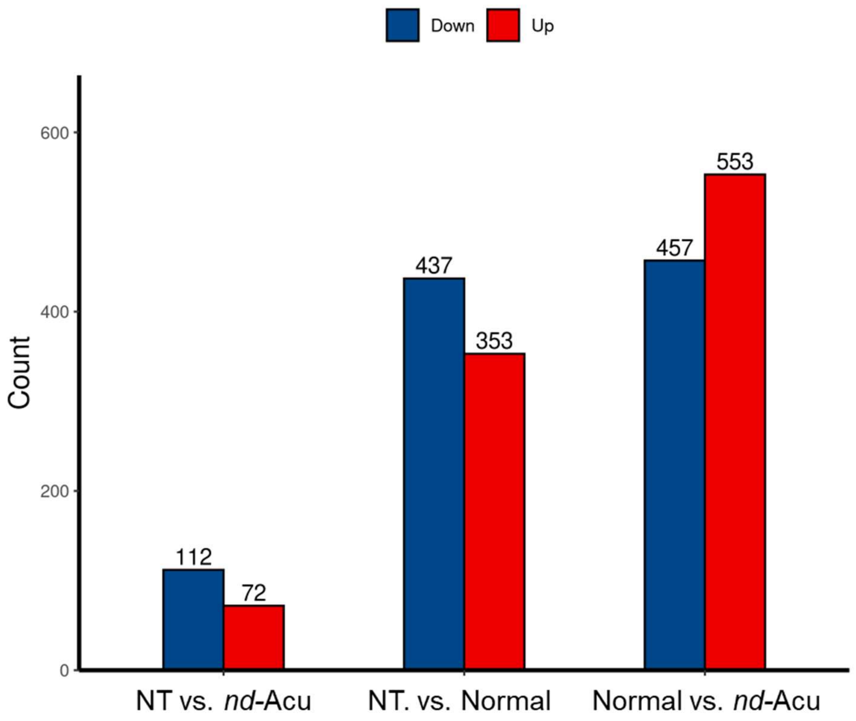
Down
Up
Count
600
400
200
0
112
72
437
353
457
553
NT vs. *nd*-Acu
NT. vs. Normal
Normal vs. *nd*-Acu
